# Genome sequence of the white-rot fungus *Irpex lacteus* F17, a type strain of lignin degrader fungus

**DOI:** 10.1186/s40793-017-0267-x

**Published:** 2017-09-12

**Authors:** Mengwei Yao, Wenman Li, Zihong Duan, Yinliang Zhang, Rong Jia

**Affiliations:** 1 School of Life Sciences, Economic and Technology Development Zone, Anhui University, 111 jiulong Road, Hefei, Anhui 230601 People’s Republic of China; 2 Anhui Key Laboratory of Modern Biomanufacturing, Anhui University, Hefei, 230601 People’s Republic of China

**Keywords:** Short genome report, Genome sequence, *Irpex lacteus* F17, White-rot fungus, Hardwood tree, Lignin decomposition

## Abstract

*Irpex lacteus*, a cosmopolitan white-rot fungus, degrades lignin and lignin-derived aromatic compounds. In this study, we report the high-quality draft genome sequence of *I. lacteus* F17, isolated from a decaying hardwood tree in the vicinity of Hefei, China. The genome is 44,362,654 bp, with a GC content of 49.64% and a total of 10,391 predicted protein-coding genes. In addition, a total of 18 snRNA, 842 tRNA, 15 rRNA operons and 11,710 repetitive sequences were also identified. The genomic data provides insights into the mechanisms of the efficient lignin decomposition of this strain.

**Electronic supplementary material:**

The online version of this article (10.1186/s40793-017-0267-x) contains supplementary material, which is available to authorized users.

## Introduction

*Irpex lacteus*, a white-rot fungus with biotechnological potential, is currently considered the most important lignocellulose-degrading organism because of its potential to degrade lignin and bioremediate other lignin-related pollutants (such as industrial dyes and aromatic pollutants) [1–3]. Lignocellulose, which is the most abundant renewable biomass in terrestrial environments, is composed of three major components: cellulose, hemicellulose, and lignin [4]. Among them, lignin is a highly irregular and heterogeneous biopolymer, which makes it recalcitrant to degradation. Compared with other wood-decay fungi, *I. lacteus* plays an important role in the efficient enzymatic conversion of renewable biomass, and it shows remarkable resistance to pollutant toxicity in water and soil environments [5]. *I. lacteus* is known to remove various aromatic compounds, including endocrine disruptors, synthetic dyes, and polycyclic aromatic hydrocarbons [1, 6], and it can also be used to obtain ethanol via the biological pre-treatment of lignocellulose [7].

*I. lacteus* is a cosmopolitan species that is widespread in Europe, North America, and Asia [8–10]. The fungus produces hydrolases, such as exo- and endo-cellulases, and extracellular oxidative enzymes, such as LiP, MnP, as well as Lac [11, 12], thereby showing a pattern of ligninolytic enzymes that is typical of white-rot fungi. Starting in the 1960’s, several studies by Japanese researchers mainly focused on the activities of the exo- and endo-cellulases, as well as an exo-cellulase gene, from *I. lacteus* [13]. Subsequently, the LiP and MnP of *I. lacteus* were isolated and characterized, and the biotechnological applicability of this fungus has drawn considerably interests in recent years [5]. Recently, we have degraded and detoxicated the synthetic dyes by using manganese peroxidase isolated from *I. lacteus* F17 [14, 15]. However, the genome sequence of *I. lacteus* has not been reported. Thus, the genomic traits of *I. lacteus* are required to reveal and elucidate the ligninolytic potential of the type strain of white-rot fungi. Here, the genome sequence of *I. lacteus* F17 is presented. To the best of our knowledge, this is the first high-quality draft genome sequence of *I. lacteus* available so far.

## Organism information

### Classification and features

**Fig. 1:**
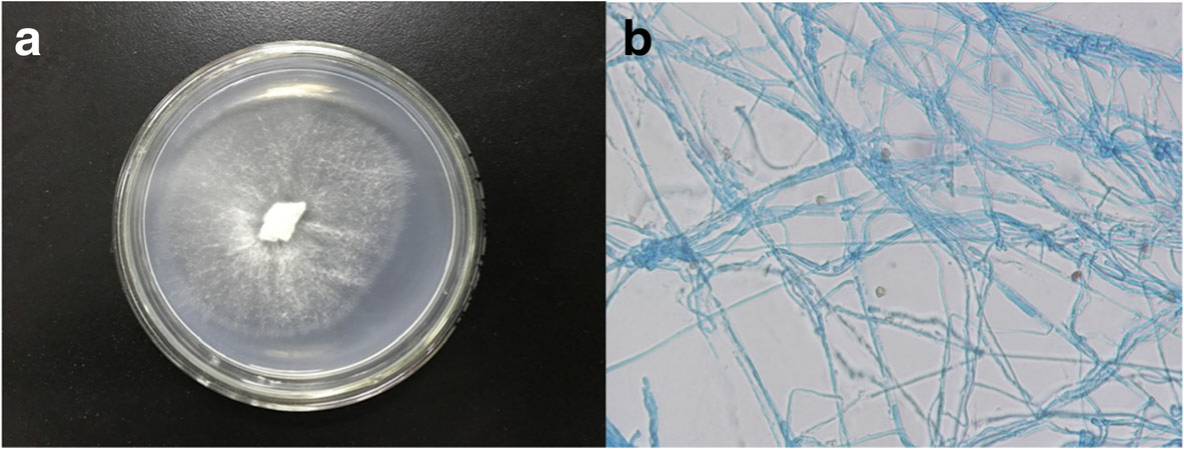
**a:** Colony of *I. lacteus* F17 grown on PDA medium for 5 days at 28 °C; **b:** Micrograph of *I. lacteus* F17 mycelia using optical microscope with 400× magnification. Mycelia were stained with lactophenol cotton blue stain solution

The sequenced strain of *I. lacteus* F17 was isolated from a decaying hardwood tree in May 2009 in the vicinity of Hefei, China (Table 1). Figure 1a shows the growth status of *I. lacteus* F17 which was cultured on PDA medium (200 g/L of potato extract, 20 g/L of glucose, and 20 g/L of agar) after 5 days at 28 °C. The strain grew faster and formed a white colony with a diameter of 6.8 cm. The micrograph of *I. lacteus* F17 mycelia grown on PDA after 3 days was obtained by OLYMPUS BX51 (Fig. 1b). The mycelia were picked up from an agar plate using a tiny tweezer, mounted on glass slides, and then stained with an appropriate amount of fungal staining solution mixed with lactic acid, carbolic acid and cotton blue (lactic acid 10 mL, carbolic acid 10 g, glycerol 20 mL, cotton blue 0.02 g, distilled water 10 mL) for light microscopic examination (400×).

**Table 1:** Classification and general features of *Irpex lacteus* F17 [19]

MIGS ID	Property	Term	Evidence code^a^
	Classification	Domain *Fungi*	TAS [5]
		Phylum *Basidiomycota*	TAS [5]
		Class *Basidiomycetes*	TAS [5]
		Order *Polyporales*	TAS [5]
		Family *Polyporaceae*	TAS [5]
		Genus *Irpex*	TAS [14]
		Species *Irpex lacteus*	TAS [14]
		Strain: F17	TAS [14]
	Gram stain	n/a	n/a
	Cell shape	Filaments	TAS [5]
	Motility	Non-motile	TAS [5]
	Sporulation	Basidiospore	NAS
	Temperature range	Not reported	n/a
	Optimum temperature	28 °C	NAS
	pH range; Optimum	Not reported	n/a
	Carbon source	Potato, Glucose	TAS [14, 15]
MIGS-6	Habitat	Dead wood, hardwood tree	TAS [5, 14]
MIGS-6.3	Salinity	Not reported	n/a
MIGS-22	Oxygen requirement	Aerobic	TAS [14, 15]
MIGS-15	Biotic relationship	Free-living	TAS [5]
MIGS-14	Pathogenicity	Not reported	n/a
MIGS-4	Geographic location	Mountain Dashu, Hefei, China	TAS [14, 15]
MIGS-5	Sample collection	May 2009	TAS [14]
MIGS-4.1	Latitude	31.85	NAS
MIGS-4.2	Longitude	117.27	NAS
MIGS-4.4	Altitude	284 m	NAS

^a^Evidence codes - IDA: Inferred from Direct Assay; TAS: Traceable Author Statement (i.e., a direct report exists in the literature); NAS: Non-traceable Author Statement (i.e., not directly observed for the living, isolated sample, but based on a generally accepted property for the species, or anecdotal evidence). These evidence codes are from the Gene Ontology project [33]

**Fig. 2:**
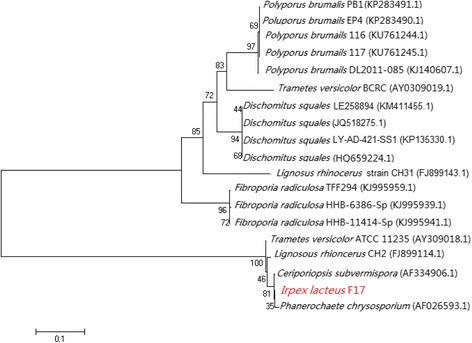
Phylogenetic tree based on 18S rRNA gene showing phylogenetic position of *I .lacteus* F17. Sequences were subjected to phylogenetic analysis using CLUSTALW [16] and MEGA 6.0 [17] to construct a nearest neighbor joining tree. The GenBank accession numbers for each strain are listed in parenthesis. The percentage of replicate trees in which the associated taxa clustered together in the bootstrap test (1000 replicates) was shown next to the branches. The GenBank accession numbers for each strain were listed in parenthesis. The tree was drawn by TreeView [18], and the scale bar represents 0.1 nucleotide substitution per nucleotide position

*I. lacteus* F17 resides in the Eukaryota, in the Fungal Kingdom, and it belongs to the family Polyporaceae, order Polyporales, class Basidiomycetes, Phylum Basidiomycota. Several other white-rot fungi with important biological function are members of the Polyporales, including *Phanerochaete chrysosporium*, *Dichomitus squalens*, *Trametes versicolor*, *Polyporus brumalis*, and *Ceriporiopsis subvermispora*. *I. lacteus* F17 has been identified and classified based on its Internal Transcribed Spacer region in our previous study [14]. The 18S rRNA gene data of *I. lacteus* F17 and several other Polyporales species were aligned using ClustalW [16]. Phylogenetic analysis based on the nearest neighbor joining method was performed using the MEGA6 package [17]. The confidence levels for the individual branches were determined by bootstrap analysis with 1000 replicates. The final phylogenetic tree was visualized with TreeView [18]. *I. lacteus* F17 is phylogenetically closely related to *C. subvermispora* (Fig. 2).

## Genome sequencing information

### Genome project history

*I. lacteus* F17 was selected for sequencing due to its bioremediation of organic pollutants and application to enzymatic biotechnologies. The genome of this strain was sequenced by SMRT technology, and genome assembly and annotation were performed at the Beijing Novogene Bioinformatics Technology Co., Ltd. (Beijing, China). The whole genome shotgun project was started in May 2016, finished in August 2016 and has been submitted to NCBI under the accession number of MQVO00000000. Table 2 summarized the project data. The project information was in compliance with MIGS version 2.0 [19].

**Table 2:** Project information

MIGS ID	Property	Term
MIGS 31	Finishing quality	High-quality draft
MIGS-28	Libraries used	Illumina:350 bp small fragment library PacBio: 20 kb SMRT Bell library
MIGS 29	Sequencing platforms	Illumina HiSeq PE150 PacBio RSII
MIGS 31.2	Fold coverage	Illumina: 20× PacBio: 70×
MIGS 30	Assemblers	SOAP denovo SMRT 2.3.0
MIGS 32	Gene calling method	PASA/Cufflinks/Augustus 2.7
	Locus Tag	BSQ47
	Genbank ID	MQVO00000000
	Genbank Date of Release	February 06, 2017
	GOLD ID	NA
	BIOPROJECT	PRJNA354901
MIGS 13	Source Material Identifier	F17
	Project relevance	Biotechnology, mycology

### Growth conditions and genomic DNA preparation

*I. lacteus* F17 was deposited at the CCTCC under the accession number of 10.1601/strainfinder?urlappend=%3Fid%3DCCTCC+AF+2014020. The strain was grown on PDA slants for 5 days at 28 °C, at which time the mycelia were scraped from the medium and lysed by liquid nitrogen grinding. The genomic DNA was extracted using the sodium dodecyl sulfate method. The harvested DNA was analyzed by agarose gel electrophoresis and purified using AMpure PB magnetic beads and then quantified by a Qubit^®^ 2.0 fluorometer (Thermo Scientific, USA). In the end, the total amount of 28 μg DNA with a final concentration higher than 50 ng/μL and a A260/A280 ratio of 1.9 was placed in dry ice and sent to the sequencing.

### Genome sequencing and assembly

A fungal survey by Illumina massively parallel sequencing technology was first used to make an evaluation for the fine mapping and assembly optimization of the fungal genome preassembling. Then the genome of *I. lacteus* F17 was sequenced by using PacBio’s SMRT technology. For the Illumina sequencing, the genome was sequenced using a single 350 bp insert genomic DNA library that was generated on a HiSeq 4000 PE150 system (Illumina, San Diego, CA, USA). For the PacBio sequencing, the genomic DNA was sheared into 20 kb fragments using a g-TUBE (Covaris, Woburn, MA, USA), and it was sequenced on an RSII system (PacBio, Menlo Park, CA, USA) after constructing the SMRT Bell library. The average sequencing depth of the 350 bp library was 20×, whereas the depth of the PacBio library was 70×.

Two assembly strategies were used respectively after filtering low-quality reads. A fungal survey produced 1564 Mb of clean data from 1700 Mb of raw data using SOAP denovo technology [20]. The PacBio subreads which were assembled into a primary assembly were completed with the Hierarchical Genome Assembly Process (Pacific Biosciences). A total of 3494 Mb of clean data were detected from the genome of *I. lacteus* F17 using samtools to fix the errors from the PacBio. The low quality reads were filtered by the SMRT 2.3.0 technology [21], and the filtered reads were assembled to generate one contig without gaps. A total of 317 contigs with an N50 of 1.15 Mb were generated from *I. lacteus* F17 genome. Finally, a 44.36 Mb draft genome of *I. lacteus* F17 was obtained. In addition, we used BUSCO [22] to assess the completeness of *I. lacteus* F17 genome and the genome has an estimated completeness of 86.9%, which indicated that we obtained a high-quality genome assembly in this study.

### Genome annotation

By combining three types of genotype calling, including de novo PASA prediction of Transdecoder/Glimmer/Snap based on transcriptome data, Cufflinks prediction based on transcriptome data and de novo Augustus (version 2.7) [23], a total number of 10,391 protein coding genes were predicted. The interspersed repetitive sequences were predicted using the RepeatMasker [24]. The tandem repeats were analyzed by the Tandem Repeats Finder [25] and the tRNA genes were predicted by the tRNAscan-SE [26]. The rRNA genes were analyzed by the rRNAmmer [27] and the snRNA were predicted by BLAST against the Rfam [28, 29] database. In the end, 18 snRNA, 842 tRNA, 15 rRNA operons and a total of 11,710 repetitive sequences were identified in the genome. Seven databases, including Gene Ontology, Kyoto Encyclopedia of Genes and Genomes, COG, Non-Redundant Protein Database, Transporter Classification Database, Swiss-Prot, and Pfam database were employed to predict gene functions. A whole genome BLAST search (E-value less than 1e-5, minimal 2 alignment length percentage larger than 40%) was performed against above seven databases. All putative proteins were compared to the entries in the CAZy database using a BLAST search. Secreted proteases were predicted with SignalP 4.1 [30] and TMHMM 2.0 [31], respectively. Other proteins that are important in wood-decay (oxidoreductases) and connected to fungal secondary metabolism were also predicted, according to a previously published method [4, 32].

## Genome properties

The draft genome sequence was based on an assembly of 317 contigs amounting to 44,362,654 bp, with a GC content of 49.64% (Table 3). From the genome, 875 RNAs (including 18 snRNA, 842 tRNA, and 15 rRNA operons), as well as 11,710 repetitive sequences, were detected. In addition, a total of 10,661 genes were predicted, of which 10,391 are protein coding genes. Table 4 presented the distribution of genes into COGs functional categories. Of the last, 2065 genes (19.37%) were assigned to COG functional categories, the most abundant of them lies in the COG category named “Posttranslational modification, protein turnover, chaperones” (245 proteins) followed by “Translation, ribosomal structure and biogenesis” (215 proteins), “General function prediction only” (211 proteins), “Energy production and conversion” (168 proteins), “Nucleotide transport and metabolism” (144 proteins), “RNA processing and modification” (121 proteins), and “Intracellular trafficking and secretion” (116 proteins).

**Table 3:** Genome statistics

Attribute	Value	% of total
Genome size (bp)	44,362,654	100.00
DNA coding (bp)	15,030,327	33.88
DNA G + C (bp)	22,021,621	49.64
DNA scaffolds	–	
Total genes	10,661	100.00
Protein coding genes	10,391	97.47
RNA genes	875	8.21
Pseudo genes	unknown	
Genes in internal clusters	unknown	
Genes with function prediction	7532	70.65
Genes assigned to COGs	2065	19.87
Genes with Pfam domains	6287	58.97
Genes with signal peptides	761	7.1
Genes with transmembrane helices	2752	25.81
CRISPR repeats	0

**Table 4:** Number of genes associated with general COG functional categories

Code	Value	% age	Description
J	215	2.07	Translation, ribosomal structure and biogenesis
A	121	1.16	RNA processing and modification
K	83	0.80	Transcription
L	56	0.54	Replication, recombination and repair
B	42	0.40	Chromatin structure and dynamics
D	63	0.61	Cell cycle control, Cell division, chromosome partitioning
V	4	0.04	Defense mechanisms
T	114	1.10	Signal transduction mechanisms
M	22	0.21	Cell wall/membrane/envelope biogenesis
N	0	0.00	Cell motility
U	116	1.12	Intracellular trafficking and secretion
O	245	2.36	Posttranslational modification, protein turnover, chaperones
C	168	1.62	Energy production and conversion
G	78	0.75	Carbohydrate transport and metabolism
E	144	1.39	Amino acid transport and metabolism
F	44	0.42	Nucleotide transport and metabolism
H	45	0.43	Coenzyme transport and metabolism
I	86	0.83	Lipid transport and metabolism
P	64	0.62	Inorganic ion transport and metabolism
Q	28	0.27	Secondary metabolites biosynthesis, transport and catabolism
R	211	2.03	General function prediction only
S	66	0.64	Function unknown
–	8374	80.59	Not in COGs

The total is based on the total number of protein coding genes in the genome

A total of 320 CAZyme-encoding genes were identified, including 53 CBMs, 161 GHs, 30 glycosyl transferases, four polysaccharide lyases, 64 AAs, and eight carbohydrate esterases (Additional file 1: Table S1). In conclusion, *I. lacteus* F17 possesses more CAZy families than other fungi (Additional file 2: Table S2), especially in the families AA3 (17 copies), AA9 (21 copies), CBM1 (34 copies), and GH5 (24 copies), which are all involved in plant cell wall degradation.

## Insights from the genome sequence

Until now, this is the first draft genome sequence of the genus *Irpex*. The phylogenetic analysis based on the 18S rRNA gene data confirms its closest relationship of *I. lacteus* F17 to *C. subvermispora*. Annotation of the *I. lacteus* F17 genome indicates that this strain possesses 320 carbohydrate-active enzymes, 191 lignin-related oxidoreductases, 568 secreted proteases, and six secondary metabolism gene clusters (Additional file 3: Table S3), all of which confirm its high lignin decomposition ability. Fifteen enzymes were classified as probable ligninolytic enzymes, including a Lac, an LiP, and 13 MnPs, one of which was identified previously [14]. Interestingly, both *I. lacteus* F17 and *C. subvermispora* have the largest number of MnPs, even greater than that of *P. chrysosporium* (five MnPs), as determined by comparing 34 basidiomycetes, including 26 fungal species belonging to the Polyporales, as well as eight species in Agaricales, Russulales, Hymenochaetales, and Corticiales, respectively (Additional file 4: Table S4). A high number of MnP isozymes suggest that *I. lacteus* F17 has a good ability to degrade lignin and other organic pollutants.

## Conclusions

In this study, we characterized the genome of *I. lacteus* F17 that was isolated from a decaying hardwood tree in the vicinity of Hefei, China. Notably, this is a first discovered sequenced strain, and we found it has lots of lignocellulose decomposition related genes. The genome sequencing information not only revealed its ligninolytic enzyme diversity, but also contributed to a better understanding of the efficient lignin decomposition of this strain. In summary, *I. lacteus* F17 has become one of model ligninolytic basidiomycetes whose availability of genomic sequences will facilitate future genetic engineering to degrade lignin and other organic pollutants.

## Additional files

Additional file 1: **Table S1.** Total CAZy families in *I. lacteus* F17. (XLSX 17 kb)

Additional file 2: Table S2. Selection of the CAZy families involved in plant cell wall degradation. (XLS 40 kb)

Additional file 3: Table S3. Gene contents in oxidoreductases, secreted proteases and secondary metabolism in the genomes of *I. lacteus* F17. (DOCX 15 kb)

Additional file 4: Table S4. Comparison of the number of MnPs from 34 fungal species belonging to the Polyporales and eight other fungi. (XLS 38 kb)

## Abbreviations

AA: Auxiliary activities
BLAST: Basic local alignment search tool
CAZy: Carbohydrate-active enzymes
CBM: Carbohydrate-binding modules
CCTCC: China Center for Type Culture Collection
COG: Clusters of orthologous groups
GH: Glycoside hydrolases
Lac: Laccase
LiP: Lignin peroxidase
MnP: Manganese peroxidase
PacBio: Pacific Bioscience
PDA: Potato dextrose agar
SMRT: Single Molecule Real-Time
