# Supplementary material for: Genome sequence of the white-rot fungus Irpex lacteus F17, a type strain of lignin degrader fungus
Source: Stand Genomic Sci. 2017 Sep 12;12:55. doi: 10.1186/s40793-017-0267-x (PMC5596461; doi:10.1186/s40793-017-0267-x)
Supplement: Supplementary file 3 — Gene contents in oxidoreductases, secreted proteases and secondary metabolism in the genomes of I. lacteus F17. (DOCX 15 kb) [file 40793_2017_267_MOESM3_ESM.docx]

**Table S3.** Gene contents in oxidoreductases, secreted proteases and secondary metabolism in the genomes of *Irpex lacteus* F17.

| Oxidoreductases | |
| --- | --- |
| MCO (multicopper oxidases) | 1 |
| CDH (cellobiose dehydrogenase) | 1 |
| CRO (copper radical oxidases) | 6 |
| POD (class II peroxidases) | 14 |
| QRD (quinone reductases) | 9 |
| HTP (heme-thiolate peroxidases) | 2 |
| DyP (dye-decolorizing peroxidases) | 5 |
| P450 (cytochrome P450) | 153 |
| OXO (oxalate oxidase/decarboxylases) | 0 |
| GLP (Fe(III)-reducing glycopeptides) | 0 |
| Cytb562 (cytochrome b562) | 0 |
| Secreted proteases | 568 |
| Secondary metabolism | |
| FAS (fatty acid synthase) | 0 |
| PKS (polyketide synthases) | 1 |
| PKS-like | 1 |
| NRPS-like(non-ribosomal peptide synthase) | 4 |
| TS (terpene synthase) | 0 |
